# The electron distribution in the “activated” state of cytochrome *c* oxidase

**DOI:** 10.1038/s41598-018-25779-w

**Published:** 2018-05-14

**Authors:** Jóhanna Vilhjálmsdóttir, Robert B. Gennis, Peter Brzezinski

**Affiliations:** 1 Department of Biochemistry and Biophysics, The Arrhenius Laboratories for Natural Sciences, Stockholm University, SE-106 91 Stockholm, Sweden; 2 Department of Biochemistry, University of Illinois at Urbana Champaign, Urbana, Illinois, 61801 United States

## Abstract

Cytochrome *c* oxidase catalyzes reduction of O_2_ to H_2_O at a catalytic site that is composed of a copper ion and heme group. The reaction is linked to translocation of four protons across the membrane for each O_2_ reduced to water. The free energy associated with electron transfer to the catalytic site is unequal for the four electron-transfer events. Most notably, the free energy associated with reduction of the catalytic site in the oxidized cytochrome *c* oxidase (state **O**) is not sufficient for proton pumping across the energized membrane. Yet, this electron transfer is mechanistically linked to proton pumping. To resolve this apparent discrepancy, a high-energy oxidized state (denoted **O**_**H**_) was postulated and suggested to be populated only during catalytic turnover. The difference between states **O** and **O**_**H**_ was suggested to be manifested in an elevated midpoint potential of Cu_B_ in the latter. This proposal predicts that one-electron reduction of cytochrome *c* oxidase after its oxidation would yield re-reduction of essentially only Cu_B_. Here, we investigated this process and found ~5% and ~6% reduction of heme *a*_3_ and Cu_B_, respectively, i.e. the apparent redox potentials for heme *a*_3_ and Cu_B_ are lower than that of heme *a*.

## Introduction

**Figure 1:**
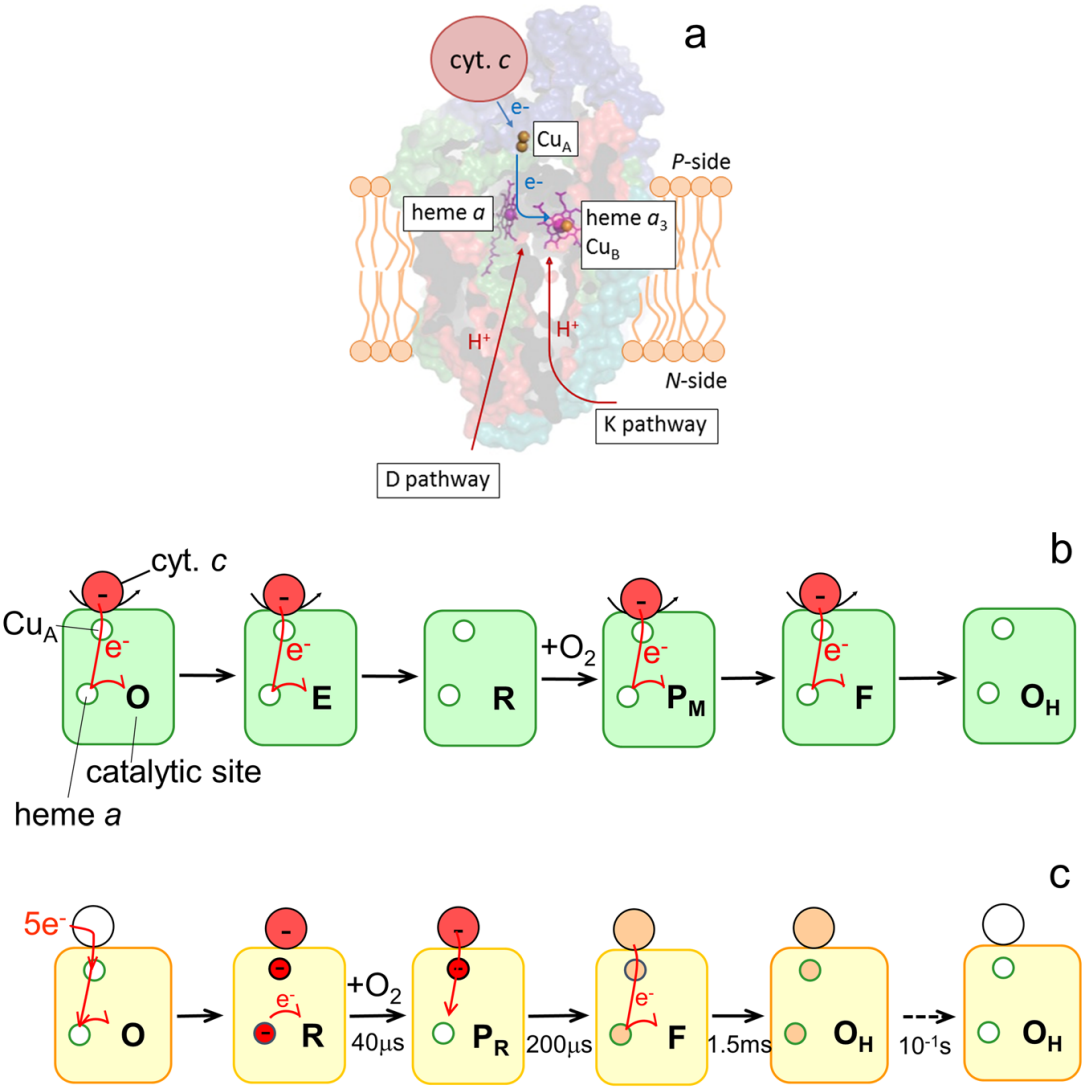
Experimental system and the studied reaction. (**a**) Cyt*c*O accommodates four redox-active centers: Cu_A_, heme *a* and the catalytic site composed of heme *a*_3_ and Cu_B_, that upon reduction each bind one electron. The cyt. *c*-Cyt*c*O electrostatic complex accommodates in total five electrons in the reduced state. Two proton-uptake pathways, called D and K are indicated in the figure, but not discussed in the text. (**b**) Turnover of Cyt*c*O. One electron at a time is donated from cyt. *c* to Cyt*c*O. The initial state is the “relaxed” oxidized state. Consecutive addition of two electrons yields the two-electron reduced catalytic site which binds O_2_ to form the peroxy state P_M_. Reduction of P_M_ yields F, which is then reduced to form the “activated” oxidized state, O_H_. This state is then reduced by cyt. *c*. (**c**) Reaction studied in this work. The oxidized cyt. *c*-Cyt*c*O complex is reduced by 5 electrons. Upon initiation of the reaction (with a laser flash, not shown), O_2_ binds to the catalytic site and an electron is transferred from heme *a* to the catalytic site to form the peroxy state, P_R_. This step is followed in time by formation of the ferryl state, F, which occurs over the same time scale as electron equilibration among Cu_A_/cyt. *c* and heme *a*. Electron transfer from this equilibrium to the catalytic site leads to formation of state O_H_. Over a longer time scale of ~0.1 s an electron equilibrates between the Cyt*c*O molecules via the bound cyt. *c* to eventually yield oxidized Cyt*c*O. Note that proton-transfer reactions are not indicated in the Figure.

The electrochemical proton gradient across the inner mitochondrial or bacterial cytoplasmic membrane in aerobic organisms is maintained by a series of membrane-bound redox-driven proton pumps. The final electron acceptor, O_2_, binds to cytochrome *c* oxidase (Cyt*c*O) at a bimetallic heme *a*_3_-Cu_B_ catalytic site where it is reduced by four electrons to water (Fig. 1a). During turnover of Cyt*c*O, electrons are transferred one-by-one from a water-soluble or membrane-associated cyt. *c* consecutively to the primary electron acceptor, Cu_A_, the secondary acceptor heme *a* and the catalytic site. The Cyt*c*O links this electron transfer to proton pumping from the more negative (*N*) to the more positive (*P*) side of the membrane with an average stoichiometry of one pumped proton per electron transferred to the catalytic site. Thus, the overall reaction is:

1 $$4{{\rm{e}}}_{{\rm{p}}}^{-}+8{{\rm{H}}}_{{\rm{N}}}^{+}+{{\rm{O}}}_{2}\to 2{{\rm{H}}}_{2}{\rm{O}}+4{{\rm{H}}}_{{\rm{p}}}^{+}$$

where the subscripts *P* and *N* refer to the two sides of the membrane. The free energy for reduction of O_2_ by cyt. *c* is ~2 eV, which is used to move 8 charges across the membrane; 4 protons that are pumped across the membrane and, 4 electrons and 4 protons that are transferred from opposite sides of the membrane resulting in a charge separation (summarized in^1^).

A number of distinct intermediate states have been identified and characterized during Cyt*c*O turnover (Fig. 1b). Consecutive transfer of two electrons to the oxidized Cyt*c*O (denoted **O**) yields formation of the one- (denoted **E**) and two- (denoted **R**) electron reduced catalytic site. When heme *a*_3_ and Cu_B_ are reduced, O_2_ binds to the heme iron after which the O-O bond is broken to form a ferryl state called “peroxy” for historical reasons (denoted **P**_**M**_). Transfer of an additional electron and a proton to **P**_**M**_, results in formation of the ferryl state, **F**. Further transfer of an electron and a proton to **F** yields the oxidized Cyt*c*O (**O**_**H**_, see below). The overall process has been reviewed in the past^1–14^ and it is summarized in Fig. 1b.

Early studies indicated that the free energy from O_2_ reduction is not released evenly in the four electron-transfer reactions, but the majority of this free energy was found to be liberated in the **P**_**M**_ → **F** and **F** → **O** reaction steps^15^. Furthermore, elegant studies performed by Wikström suggested that proton pumping is coupled only to these two steps^16^, which stimulated Wikström and colleagues to propose a detailed molecular model based on these findings^17^. According to this model, two protons would be pumped in each of the reactions **P**_**M**_ → **F** and **F** → **O**, while no proton pumping would be observed upon reduction of the Cyt*c*O, i.e. **O** → **E** → **P**_**M**_. Later, a novel kinetic approach was used to obtain data^18^ that resulted in re-evaluation of the model to yield a scenario where one proton is pumped in each of the four reaction steps that involve electron transfer to the catalytic site^19^.

A key aspect of the new model was postulation of a “high-energy” metastable oxidized form of the Cyt*c*O that would presumably differ in structure from the oxidized “as isolated” (**O**) state. The state is denoted **O**_**H**_ and it is assumed to be formed as a product of the **F** → **O**_**H**_ reaction (below we denote by **O**_**H**_ the oxidized state that is formed after re-oxidation of the reduced Cyt*c*O independently of the properties of this state)^18^. According to this scenario part of the free energy released in **P**_**M**_ → **F** and **F** → **O**_**H**_ would be conserved in the **O**_**H**_ state to be used during subsequent reduction of the Cyt*c*O, i.e. the reaction sequence **O**_**H**_ → **E** → **P**_**M**_ would be more exergonic than **O** → **E** → **P**_**M**_. The question arises: what is the difference between states **O** and **O**_**H**_?

Postulation of state **O**_**H**_ also found support in an experimental observation: when starting from the fully oxidized Cyt*c*O (**O**) the free energy associated with electron transfer from cyt. *c* to heme *a*_3_-Cu_B_, before O_2_ binds, is too small to drive proton translocation (see Kaila *et al*.^1^). Consequently, one possible difference between the “as isolated” state **O** and state **O**_**H**_ could be that in the latter the midpoint potentials of heme *a*_3_ and Cu_B_ are higher than those in state **O**^19,20^. Indeed, data from experimental^21^ and theoretical^22^ studies suggested that the midpoint potential of Cu_B_ is significantly elevated in state **O**_**H**_. Furthermore, Blomberg and Siegbahn found that the inherent midpoint potential of Cu_B_ is high during catalytic turnover, and that in the resting oxidized state a slow protonation process may cause a significant decrease of the proton-coupled reduction potential^23^. As discussed in the paper by Belevich *et al*.^21^, there are conflicting experimental data regarding possible differences in the Cu_B_ midpoint potentials between states **O** and **O**_**H**_. For example, Jancura *et al*.^24^ did not observe any spectral or kinetic differences between the **O**_**H**_ and **O** forms of Cyt*c*O. Brand *et al*.^25^ prepared Cyt*c*O in the **O** and **O**_**H**_ states, respectively, and then added one electron to each of the samples. They found that the extent of electron transfer from heme *a* to the catalytic site was relatively small and the same in both states, which indicated that the midpoint potential of Cu_B_ was not raised in state **O**_**H**_.

In the present study, we investigated the reaction of the reduced *Rhodobacter sphaeroides* Cyt*c*O with O_2_ in the absence and presence, respectively, of cyt. *c* at low ionic strength. In the latter case a cyt. *c*-Cyt*c*O electrostatic complex is formed (see Fig. 1a,c). This arrangement contrasts the earlier studies in that it resembles the *in vivo* situation where electrons are continuously fed into Cyt*c*O from the donor cyt. *c*. The cyt. *c*-Cyt*c*O complex accommodates five electrons. Upon binding of O_2_ four electrons are transferred to form H_2_O yielding the oxidized Cyt*c*O, presumably in state **O**_**H**_. Then the “fifth electron” can equilibrate among the five sites cyt. *c*, Cu_A_, heme *a*, heme *a*_3_ and Cu_B_^26–28^. We reasoned that if in state **O**_**H**_ the Cu_B_ midpoint potential would be elevated, this fifth electron would be transferred to Cu_B_. The data show that this was not the case and in ~90% of Cyt*c*Os the fifth electron was distributed among sites cyt. *c*, Cu_A_ and heme *a* with the largest fraction at heme *a*.

## Results and Discussion

### Spectral analysis

**Figure 2:**
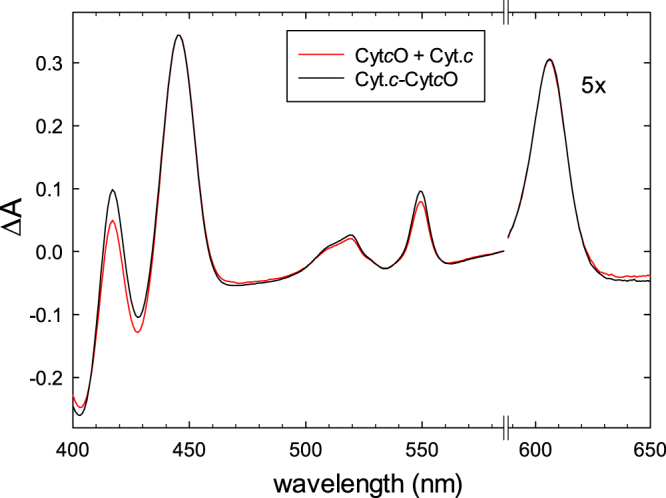
Absorbance difference spectra. A comparison of reduced minus oxidized difference spectra of the Cyt*c*O-cyt. *c* complex (black) and the sum of the reduced minus oxidized difference spectra of Cyt*c*O and cyt. *c* (red), respectively. Spectra of the oxidized states were recorded first and then the atmosphere in the cuvette was replaced for N_2_ after which the samples were reduced with ascorbate (2 mM) and hexaammineruthenium(II) chloride (1 μM). Reduction of the samples was followed in time by recording spectra over ~2 hours until no further changes were observed. The data to the right of the axis break have been multiplied by a factor of five. The concentrations of Cyt*c*O and cyt. *c* were ~3 μM and ~5 μM, respectively, in 50 mM HEPES (pH 7.5), 0.05% DDM and 100 µM EDTA.

To determine whether or not binding of cyt. *c* to Cyt*c*O affects the spectral properties of the hemes, we recorded the reduced - oxidized difference spectra of each of the isolated proteins (cyt. *c* and Cyt*c*O) as well as of a mixture of the two at low ionic strength (i.e. the cyt. *c*-Cyt*c*O complex). As seen in Fig. 2, the sum of the cyt. *c* and Cyt*c*O difference spectra was essentially equal to that of the mixture, except for a small increase in the 550 nm absorbance peak upon forming the cyt. *c*-Cyt*c*O complex.

### Reaction of Cyt*c*O with O_2_

**Figure 3:**
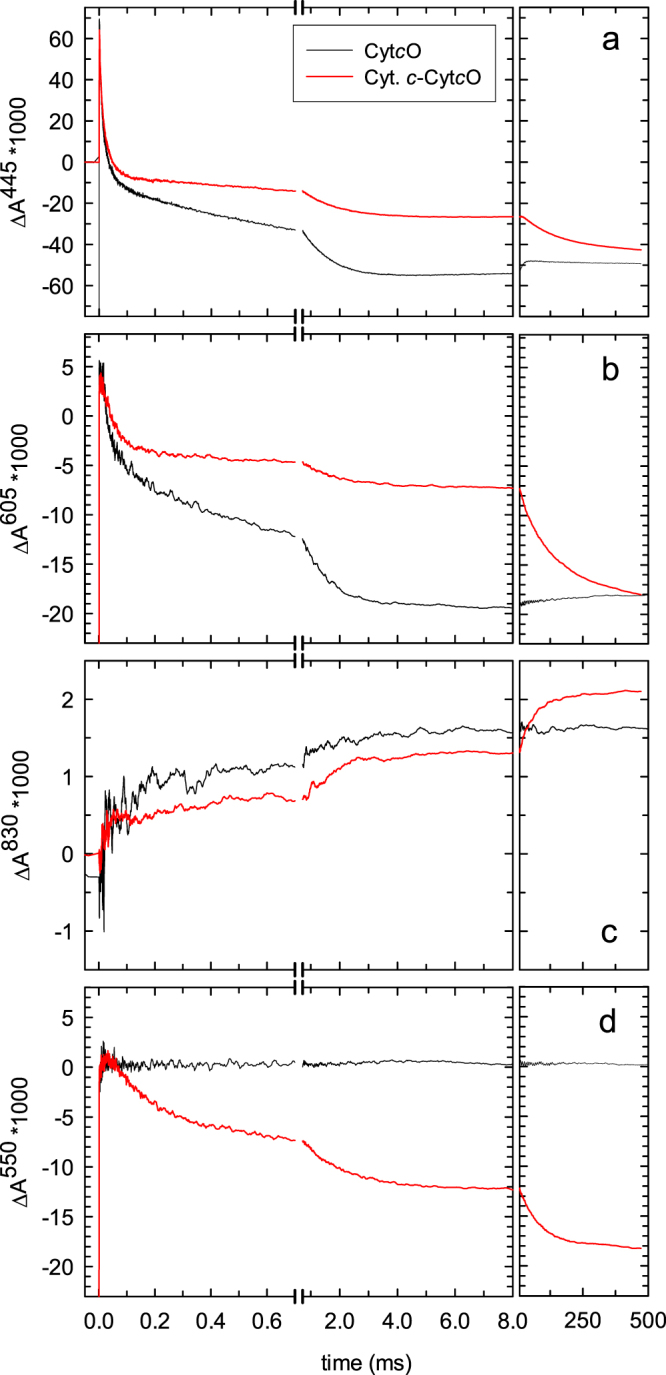
Kinetics of absorbance changes upon reaction with O_2_. The four-electron reduced Cyt*c*O (black traces) or the five-electron-reduced cyt. *c*-Cyt*c*O complex (red traces) was mixed with an O_2_-saturated buffer solution. About 200 ms after mixing with the O_2_-containing buffer, at time = 0, the CO-ligand was removed by a laser flash. The four-electron complex becomes oxidized over a time scale of 8 ms (left-hand side boxes). At 445 nm (**a**) the main contribution is from redox changes at hemes *a* and *a*_3_. At 605 nm (**b**) the main contribution is from redox changes at heme *a* (80%) and the remaining fraction originates from changes at heme *a*_3_. At 830 nm (**c**) the main contribution is from Cu_A_ where an increase in absorbance is associated with oxidation. At 550 nm (**d**) the main contribution is from redox changes at cyt. *c*. At 445 nm and 605 nm, the rapid change in absorbance at *t* = 0 is associated with CO dissociation. It is followed in time by a decrease in absorbance associated with binding of O_2_ (τ ≅ 10 μs), formation of the **P**_**R**_ state (τ ≅ 40 μs) and oxidation of the Cyt*c*O (τ ≅ 1.5 ms). The **P**_**R**_ → **F** reaction is not seen at these wavelengths. At 830 nm two components are seen with time constants of 200 µs (**P**_**R**_ → **F**) and 1.5 ms (**F** → **O**). Oxidation of cyt. *c* occurs over the same time scale. In the presence of cyt. *c* absorbance changes attributed to the Cyt*c*O (panels a–c) were smaller because the redox sites were re-reduced by cyt. *c* during the course of the reaction. Over a time scale of ~500 ms (right-hand side boxes) all redox sites become oxidized. The small increase in absorbance over this time scale in the absence of cyt. *c* is due to small fractional re-reduction of Cyt*c*O by ascorbate. Experimental conditions after mixing: 1.1 μM Cyt*c*O, 20 mM HEPES at pH 7.5, 0.05% DDM, 100 μM EDTA, 2 mM ascorbate, 1 μM hexa-ammine-ruthenium(II) chloride, 1 mM O_2_ at ~22 °C (black trace). The red traces are averages of three traces obtained under the following conditions: [cyt. *c*]/[Cyt*c*O] in μM (*i*) 2.1/1.6, (*ii*) 1.5/1.3, (*iii*) 1.7/1.3. The mixing ratio was 1:5 with an oxygen-saturated buffer solution (20 mM HEPES at pH 7.5). All traces have been scaled to 1 μM reacting Cyt*c*O based on the rapid change in absorbance at 445 nm at *t* = 0. A laser artifact at *t* = 0 has been truncated for clarity.

The reduced Cyt*c*O carries four electron equivalents. Thus, upon reaction with O_2_ the Cyt*c*O becomes fully oxidized while O_2_ is reduced to H_2_O. The anaerobic samples were mixed with an O_2_-saturated solution in a stopped-flow apparatus. After ~200 ms the CO ligand was dissociated by means of a short laser flash, which initiates the reaction of the reduced Cyt*c*O with O_2_. In the absence of cyt. *c* (black traces in Fig. 3) the reaction exhibits a number of distinct kinetic steps, while populating states in which O_2_ is progressively reduced and protonated to eventually form H_2_O. The details of the reaction sequence have been described in the past^1,4,5,29,30^ and it is outlined in Fig. 1c.

At 445 nm and 605 nm the main contribution to the absorbance changes is from both hemes *a* and *a*_3_. At 830 nm and 550 nm the main contribution is from Cu_A_ and cyt. *c*, respectively. The **A** → **P**_**R**_ and **F** → **O** reactions are seen as a decrease in absorbance with time constants of ~40 μs and ~1.5 ms (Fig. 3a,b), respectively, at both 445 nm and 605 nm. The **P**_**R**_ → **F** reaction is not seen at these wavelengths, but fractional oxidation of Cu_A_ that is linked in time to the **P**_**R**_ → **F** reaction^31^ is seen at 830 nm where we observed an increase in absorbance with a time constant of ~200 μs (Fig. 3c). Also the remaining oxidation of Cu_A_, linked in time to the **F** → **O**_**H**_ reaction, is seen as a further increase in absorbance at 830 nm. We did not observe any absorbance changes at 550 nm in the absence of cyt. *c*, which shows that at this wavelength the contribution from redox changes of Cyt*c*O is negligible (Fig. 3d, black trace).

Over a longer time scale we observed a slight re-reduction of hemes *a* and *a*_3_, seen as a small increase in absorbance at 445 nm and 605 nm (see right-hand side boxes of panels a and b).

### Reaction of the cyt. *c*-Cyt*c*O complex with O_2_

A mixture of cyt. *c* and Cyt*c*O, in a low-ionic strength solution (see Materials and Methods), was reduced and incubated under an atmosphere of CO. The cyt. *c* was added at a slight excess to Cyt*c*O (1.2–1.3 cyt. *c*/Cyt*c*O) to assure formation of the cyt. *c*-Cyt*c*O complex in the major fraction of the population (see below). We first discuss the absorbance changes over a time scale of 8 ms (black traces in Fig. 3). Over this time scale four electrons are delivered from Cyt*c*O to O_2_, as discussed above.

When cyt. *c* is bound to Cyt*c*O an electron is transferred from cyt. *c* to Cu_A_ upon the first, fractional oxidation of Cu_A_, i.e. during the **P**_**R**_ → **F** reaction (τ ≅ 200 μs), and then during the **F** → **O**_**H**_ reaction (τ ≅ 1.5 ms) while Cu_A_/heme *a* are oxidized further (red traces in Fig. 3)^26–28^. These oxidation reactions are seen as a decrease in absorbance at 550 nm with time constants of ~200 μs and ~1.5 ms, respectively. All other absorbance changes over a time scale of 8 ms were diminished in the presence of cyt. *c*, reflecting re-reduction of the Cyt*c*O redox sites by cyt. *c* (compare the red and black traces in Fig. 3a–c).

The absorption coefficients of the redox sites at the measured wavelengths are listed in Table 1. All data in Fig. 3 were scaled to 1 μM reacting Cyt*c*O, based on the absorption coefficient for the CO-dissociation absorbance change at 445 nm. This normalization was only done to simplify comparison of the data at different wavelengths and it does not influence the analysis below.

**Table 1:** Reduced minus oxidized difference absorption coefficients (Δε) of the redox sites, except the first line where the Δε is given for CO binding to the reduced heme *a*_3_.

Redox site or reaction	Δε mM^−1^cm^−1^	wavelength (nm)	Reference
Cyt*c*O-CO-Cyt*c*O	67	445	^33^
cyt. *c*	21.1	550	^46^
Cu_A_	2.3	830	^47^
	1.6		^34^
heme *a*_3_	112	445	^33^
	82.3	444	^32^
heme *a*	57	445	^33^
	66.4	446	^32^
heme *a*	20.5	605	^33^
	18.6		^32^
heme *a*_3_	4.8	605	^33^
	4.6		^32^

### Further oxidation of the cyt. *c*-Cyt*c*O complex after reaction with O_2_

In the five-electron cyt. *c*-Cyt*c*O system the single remaining electron after oxidation of the Cyt*c*O is not rapidly transferred to the excess oxygen (i.e. to a second O_2_) because O_2_ does not bind to the catalytic site when it is reduced by only one electron. Instead, oxidation of the hemes occurred over a longer time scale of ~500 ms, see right-hand side boxes in Fig. 3. This process may involve either (*i*) transfer of the electron to the optically silent Cu_B_, or (*ii*) electron exchange between the Cyt*c*O molecules, via cyt. *c*, eventually yielding a thermodynamically stable state. The oxidation state of the Cyt*c*O catalytic site in this stable state would depend on the number of electrons transferred to the specific enzyme molecule. The most likely scenario is that an electron is exchanged between two Cyt*c*Os such that one molecule becomes oxidized and the other forms state **P**_**M**_.

**Figure 4:**
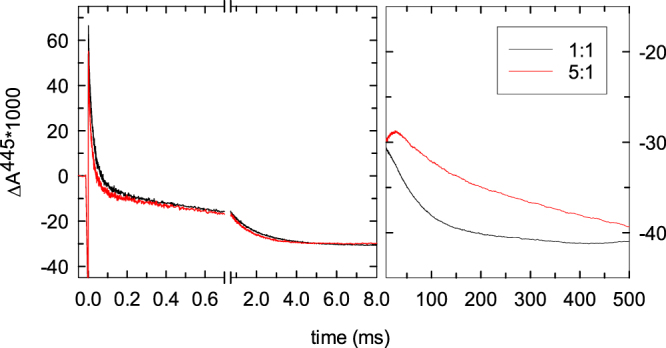
Kinetics of absorbance changes upon reaction with O_2_ upon dilution. The experiment is the same as that shown in Fig. 3, but the Cyt*c*O-containing sample was mixed 1:1 (black) or 1:5 (red) with the O_2_-containing buffer. Note the different absorbance scales in the left and right parts of the graph, respectively. The concentrations of Cyt*c*O and cyt. *c* were 5 µM and 7.5 µM, respectively.

To discriminate between these two scenarios we reasoned that in case (*i*) the time constant of the slowest oxidation reaction would be independent of the Cyt*c*O concentration while in case (*ii*) this time constant would decrease upon dilution because the reaction involves electron exchange between Cyt*c*O molecules mediated via cyt. *c*. As seen in Fig. 4, the time constant of the slowest component increased from ~75 ms to ~300 ms upon increasing the dilution of the Cyt*c*O-cyt. *c* sample with the O_2_-containing buffer from 1:1 to 1:5. This observation indicates that the slowest component is due to electron exchange between Cyt*c*O molecules according to scenario (*ii*).

At 550 nm we observed a decrease in absorbance in the time range 8-500 ms (see Fig. 3d) that was added on top of the oxidation of the fraction reduced cyt. *c* in the cyt. *c*-Cyt*c*O complex. This signal was observed because there was an excess of 1.2–1.3 cyt. *c* compared to Cyt*c*O. Over this longer time scale this excess cyt. *c* in solution, became fully oxidized.

For the Cu_A_ absorbance changes at 830 nm (Fig. 3c) the absorbance reached a higher level with than without cyt. *c* at 500 ms, which is discussed below.

### Analysis of the data

We define the absorbance difference, at wavelength λ, after *x* ms and immediately after the flash, i.e. at time = 0^+^: Δ*A*^λ^(*x*) = *A*^λ^(*t* = *x* ms) - *A*^λ^(*t* = 0^+^). As already indicated above, in the absence of cyt. *c*, Δ*A*^λ^(8) reflects oxidation of the reduced Cyt*c*O. We note that a comparison of this absorbance change at e.g. 445 nm and 605 nm, and that associated with CO-dissociation typically deviates from the equivalent differences seen in static spectra (Table 1). Therefore, we found that the most reliable approach to analyze the absorbance changes associated with oxidation of hemes *a* and *a*_3_ is to compare Δ*A*^445^(8) and Δ*A*^605^(8) with and without cyt. *c* added. This approach does not require the knowledge of absorption coefficients of hemes *a* and *a*_3_, only their relative contribution must be known. As seen in Table 1, at 605 nm hemes *a* and *a*_3_ contribute with 80% and 20%^32,33^, respectively. At 445 nm we used the relative contributions determined by Vanneste^33^: 66% and 34% for hemes *a*_3_ and *a*, respectively. The relative contributions determined by Liao *et al*.^32^ differed from these values, but the use of these values yields the same conclusions (see below).

The fractions hemes *a* and *a*_3_ (δ(*a*) and δ(*a*_3_), respectively) that become oxidized after 8 ms in the presence of cyt. *c* are determined from:

2 $$\frac{{\rm{\Delta }}{A}_{+{\rm{cyt}}.c}^{445}(8)}{{\rm{\Delta }}{A}^{445}(8)}=0.34{\rm{\delta }}(a)+0.66\,{\rm{\delta }}({a}_{3})=0.75$$

3 $$\frac{{\rm{\Delta }}{A}_{+{\rm{cyt}}.c}^{605}(8)}{{\rm{\Delta }}{A}^{605}(8)}=0.80\delta (a)+0.20\delta ({a}_{3})=0.50$$

which yields δ(*a*) = 0.40 ± 0.04 and δ(*a*_3_) = 0.94 ± 0.06 (SD, 3 measurements). In other words, ~60% of heme *a*, but only ~6% of heme *a*_3_ become re-reduced when cyt. *c* is present. When using the relative contributions of hemes *a* and *a*_3_, respectively, based on the absorption coefficients determined by Liao *et al*.^32^ (see Table 1), the corresponding values would be δ(*a*) ≅ 0.5 and δ(*a*_3_) ≅ 1, i.e. ~50% re-reduced heme *a* and no heme *a*_3_ re-reduced.

Next, we calculate the fraction Cu_A_ that becomes re-reduced in the presence of cyt. *c* from the absorbance changes at 830 nm (Fig. 3c). At this wavelength oxidation of Cu_A_ leads to an increase in absorbance.

We compare the absorbance levels after 8 ms obtained in the absence and presence of cyt. *c*: $\frac{{\rm{\Delta }}{A}_{+{\rm{cyt}}.c}^{830}(8)}{{\rm{\Delta }}{A}^{830}(8)}=$ $0.85\pm 0.08$ (SD, three measurements), which yields ~15% re-reduced Cu_A_ when cyt. *c* is present.

We note that the absorbance levels at 830 nm after 500 ms in the presence and absence of cyt. *c* differ. This difference is most likely due to contribution of state **P**_**M**_, at 830 nm^34^, formed at the catalytic site after electron exchange between the Cyt*c*Os in the presence of cyt. *c* (see explanation above).

The fraction oxidized cyt. *c* is calculated from the absorbance changes at 550 nm (Fig. 3d). We calculated the fraction from the ratio of absorbance changes at 500 ms (full oxidation of the cyt. *c* pool) and 8 ms, respectively, assuming that the fraction oxidized cyt. *c* over the time scale of 8 ms reflects cyt. *c* that was bound in complex with Cyt*c*O.

The fraction oxidized cyt. *c* after 8 ms, $\frac{{\rm{\Delta }}{A}_{+{\rm{cyt}}.c}^{550}(8)}{{\rm{\Delta }}{A}_{+{\rm{cyt}}.c}^{550}(500)}$ is 0.68. This number must be scaled for the molar excess cyt. *c* as compared to Cyt*c*O, which is 1.2 (in the experiment shown in Fig. 3d). Thus, assuming a 1:1 cyt. *c*-Cyt*c*O complex, the absorbance changes at 8 ms yield oxidation of 84 ± 7% (SD, 3 measurements) of the bound cyt. *c*. The occupancy of the cyt. *c*-binding site is not known, but because the degree of cyt. *c* oxidation is close to unity, the assumption of a 1:1 cyt. *c*:Cyt*c*O complex is at least approximately correct.

**Figure 5:**
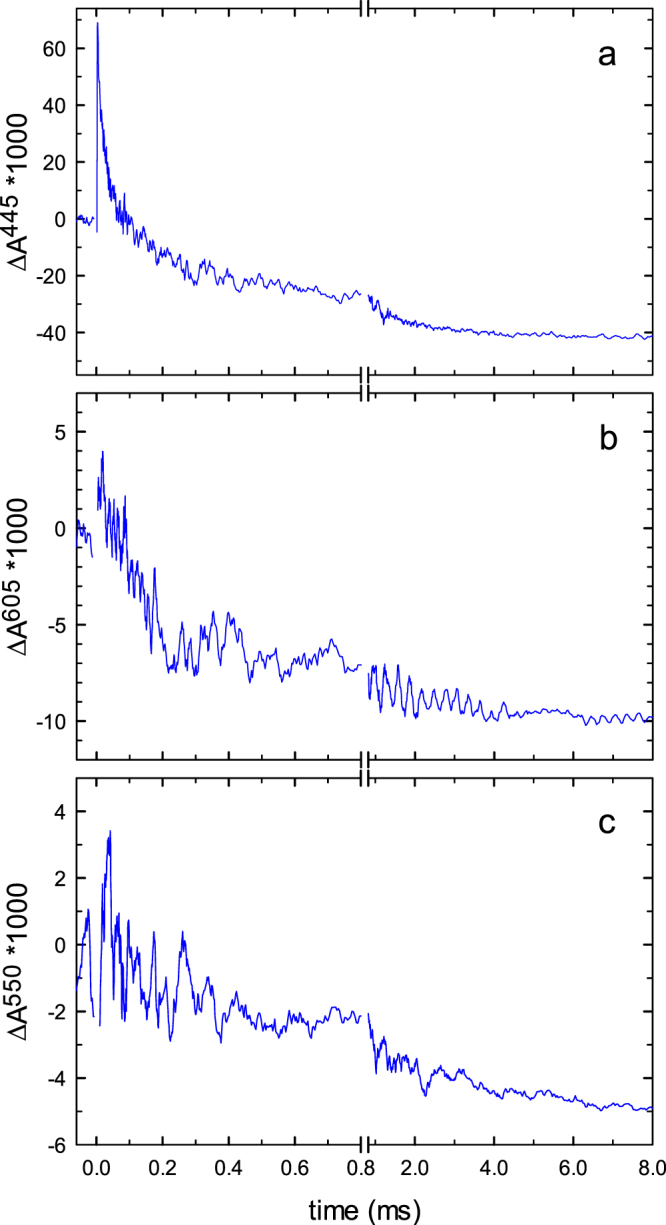
Reaction of membrane fragments with O_2_. The experiment is the same as that shown in Fig. 3. Absorbance changes were monitored at 445 nm (**a**) 605 nm (**b**) and 550 nm (**c**) Experimental conditions after mixing: *R. sphaeroides* membranes (∼2 µM Cyt*c*O, calculated from the reduced minus oxidized difference spectrum) in 20 mM HEPES (pH 7.5), 100 µM EDTA and 1 mM O_2_ at ∼22 °C. The mixing ratio was 1:1 with an oxygen-saturated buffer (20 mM HEPES pH 7.5). The traces have been scaled to 1 µM reacting Cyt*c*O. A laser artifact at *t* = 0 has been truncated for clarity.

To test whether or not the presence of detergent could alter the midpoint potentials of Cyt*c*O in state **O**_**H**_, we also repeated the experiments with native membranes from *R. sphaeroides* (Fig. 5). Due to light scattering of the membrane fragment the signal-to-noise ratio in these measurements was much smaller than that seen with detergent-solubilized Cyt*c*O. Therefore, we had to exclude data at 830 nm where the absorbance changes associated with redox changes at Cu_A_ are very small (see Table 1). We observed a smaller degree of cyt. *c* oxidation over ~8 ms, presumably because part of cyt. *c* binds to the membrane fragments, being inaccessible to Cyt*c*O. Nevertheless, we could estimate the degree of cyt. *c* oxidation and reduction of hemes *a* and *a*_3_. The fraction oxidized cyt. *c* per Cyt*c*O was ~0.4 (Fig. 5c), based on a comparison with the data in Fig. 3d. The fraction reduced hemes *a* and *a*_3_ was estimated using Eqs (1 and 2) above and the absorbance differences Δ*A*^445^(8) and Δ*A*^605^(8) in Fig. 5a,b. These data indicate that at 8 ms after initiation of the reaction, heme *a*_3_ is not reduced while heme *a* is essentially fully reduced, which is qualitatively consistent with the data obtained with Cyt*c*O in detergent solution.

**Figure 6:**
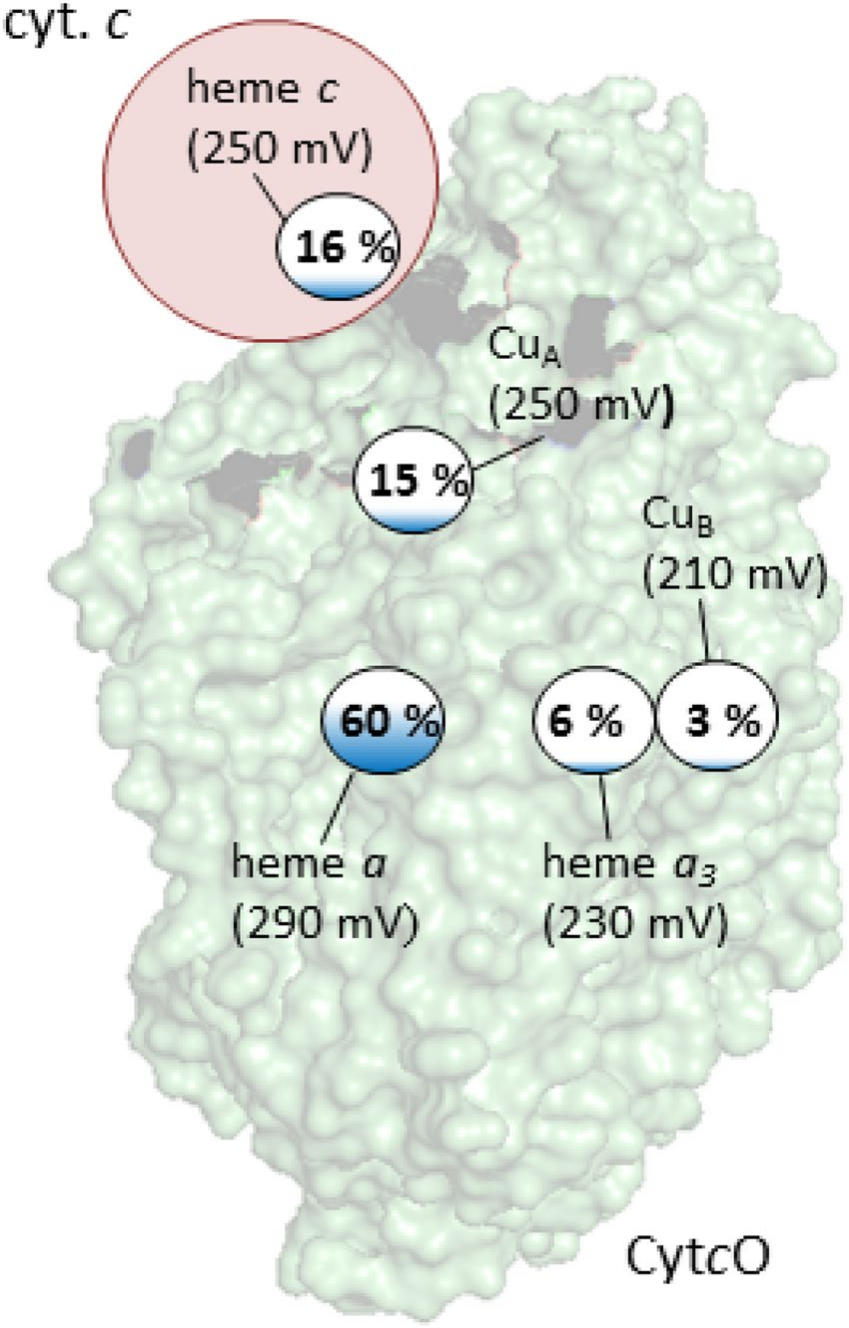
Summary of the data. The redox state of the co-factors is indicated with color. The fraction color in the small circles reflects the degree of reduction of each redox site. The apparent redox potentials were estimated from the fractions reduced redox sites assuming a midpoint potential of 250 mV for Cu_A_. The numbers are based on the assumption that the cyt. *c*-Cyt*c*O complex accommodates five redox sites and that each site can accommodate one electron. No interactions between the redox sites are considered (see text).

Interpretation of the data obtained with detergent-solubilized Cyt*c*O is summarized in the illustration in Fig. 6. We found that at the end of the initial oxidation of the Cyt*c*O, i.e. after 8 ms, the fraction reduced cyt. *c* is 0.16. In other words, we assume that 0.84 electrons reside among the four redox sites of Cyt*c*O. The reduction levels of hemes *a* and *a*_3_ were 0.60 and 0.06, respectively, while that of Cu_A_ was 0.15. These numbers are obtained from the measured absorbance changes. When adding these fractions reduced hemes *a* and *a*_3_ and Cu_A_ we obtain 0.81. Because 0.84 electrons reside at Cyt*c*O (based on the degree of oxidation of cyt. *c*), the fraction reduced Cu_B_ is 0.03. It should be noted that this number is not measured directly because Cu_B_ is optically silent. It is calculated based on the assumption that the cyt. *c*-Cyt*c*O complex comprises five redox-active centers and that each of these sites can harbor one electron.

On the basis of this distribution of the fifth electron among the five redox sites cyt. *c*, Cu_A_, heme *a*, heme *a*_3_ and Cu_B_, and assuming a midpoint potential of 250 mV for Cu_A_^35^, we estimate the apparent redox potentials of the redox sites in Cyt*c*O in state **O**_**H**_ (Fig. 6). We refer to “apparent” redox potentials because the analysis reflects only the distribution of a single electron after 500 ms in the five-electron system (among five sites). Because interactions between the redox sites are not considered (see^35,36^), the values differ from those obtained in redox titrations, which typically yield a higher midpoint potential for the catalytic site heme (here heme *a*_3_) than for the intermediate electron acceptor (here heme *a*)^35,36^, although in many bacterial oxidases the difference between the midpoint potentials is reversed^37^. Furthermore, because a single electron equilibrates among the five redox sites, the situation differs from a redox titration^38^. The purpose of the exercise to calculate the apparent redox potentials is not to provide the values, only to indicate that the apparent redox potential of Cu_B_ is not higher than that of any other site after reaction of the Cyt*c*O with O_2_. The main conclusion is that the fifth electron is not found at the catalytic site.

Belevich and colleagues^21^ concluded that upon injection of a single electron to state **O**_**H**_ less than 2% heme *a* was reduced and that the midpoint potential of Cu_B_ was raised to a value at least 100 mV higher than that of heme *a*. This contrasts the 60% reduced heme *a* observed here and we did not find an increased midpoint potential of Cu_B_. One difference between our study and that of Belevich *et al*. is the different experimental approach; we used cyt. *c* as the electron donor while Belevich *et al*. used a Ru-complex excited by light. Even though the binding geometry and overall charge of these two electron donors differ, the differences are not likely to explain the discrepancy in the Cu_B_ midpoint potentials because of the significant distance between the site where the electron donor binds and Cu_B_. Belevich and colleagues^21^ discussed the discrepancy between their results and those obtained in other laboratories prior to the present study, and suggested that they may originate from exposure of the Cyt*c*O to detergent or removal of phospholipids during enzyme isolation. As discussed above, we did not find any additional reduction of the catalytic site with Cyt*c*O in its native lipid environment.

In earlier studies using an identical experimental approach to that used here^39,40^ we did observe differences between Cyt*c*O that was reconstituted in liposomes and in detergent. However, these differences were mainly observed for the kinetics of reactions that are associated with proton uptake from solution, presumably due to interaction of lipids with groups near the entry point of the Cyt*c*O proton pathways^40^.

The experimental approach used here reflects the conditions in the native system where cyt. *c* is the electron donor. Yet, one may argue that in our experiments the electron from cyt. *c* was transferred over a time scale that is shorter than that of **O**_**H**_ formation, i.e., an alternative explanation is that the state formed is not **O**_**H**_. State **O** is defined as the “relaxed form” of the oxidized Cyt*c*O, reached after long time without turnover, i.e. not the transient state visited in the experiments described here. Consequently, if we assume that **O**_**H**_ is not formed here, we would have to assume a third oxidized state. Furthermore, we would have to postulate that formation of **O**_**H**_ requires a delay between oxidation and re-reduction of the Cyt*c*O. However, the concentration of O_2_ in mammalian tissues is only a few μM^41^, which suggests that at steady state heme *a*/Cu_A_ would be at least partly reduced, resembling the states visited in the current study, as also indicated in^42^. We also note that in the experiments described by Belevich and colleagues^21^ the delay between oxidation and injection of the fifth electron was ~5 ms and over this time scale we did not observe any additional heme oxidation.

## Summary

An activated form of the oxidized Cyt*c*O, **O**_**H**_, was postulated. The difference between states **O** and **O**_**H**_ was suggested to be an elevated midpoint potential of Cu_B_ in the latter. In the present study, we used an alternative experimental technique to approach the problem and found that in the **O**_**H**_ state the electron resides mainly at heme *a* and Cu_A_. In other words the Cu_B_ midpoint potential was not elevated in **O**_**H**_. A similar behavior was observed in native membrane, which excludes the possibility that the behavior is due to removal of lipids from the protein.

## Materials and Methods

### Preparation of *R. sphaeroides* CytcO

The *R. sphaeroides* bacteria were grown aerobically in the dark at 30 °C in a Sistrom medium. After harvesting, the cells were re-suspended in 50 mM Tris-buffer, pH 8.0 (0.2 g/ml) and stirred for one hour at 4 °C in the presence of DNase I (0.05 mg/ml final concentration). The suspension was subsequently passed twice through a continuous-flow cell disruptor (Constant Systems LTD) operating at 170 MPa. Unbroken bacteria and cell debris were removed from the suspension by low-speed ultra-centrifuging (7800 g for 15 min at 4 °C) followed by high-speed ultra-centrifugation (138 000 g for 90 minutes at 4 °C) to collect the membrane fraction. The membrane pellet was re-suspended (homogenized) in 50 mM Tris-buffer.

For purification of the Cyt*c*O the membrane fraction of the cells was solubilized in 1.5% *n*-dodecyl β -D-maltoside (DDM). The histidine-tagged Cyt*c*O was purified using Ni^2+^-NTA affinity chromatography as described previously^43,44^.

### Measurement of the oxidation kinetics

For studies of Cyt*c*O in the native membrane environment the *R. sphaeroides* membranes were diluted to ~5 μM Cyt*c*O in 20 mM HEPES (pH 7.5) and 100 μM EDTA.

For studies of the purified Cyt*c*O, the buffer-solution of the enzyme was exchanged for 20 mM HEPES (pH 7.5), 0.05% DDM and 100 μM EDTA using a gel filtration column (PD-10, GE Healthcare), resulting in a final Cyt*c*O concentration of ~10 μM. The same method was used to prepare the cyt. *c*-Cyt*c*O complex where equine heart cyt. *c* (Sigma-Aldrich) was added to the Cyt*c*O prior application to the PD-10 column. For the *R*. *sphaeroides* membrane fraction, the equine heart cyt. *c* was added directly to the sample.

The cyt. *c*-Cyt*c*O ratio was 1.2–1.3 (see text and figure legends). The sample was transferred to a locally modified Thunberg cuvette after which air in the cuvette was exchanged for N_2_ using a vacuum line. The sample was reduced upon anaerobic addition of ascorbate (2 mM) and the redox mediator hexa-ammine-ruthenium(II) chloride (1 μM). After full reduction, the N_2_ atmosphere in the cuvette was replaced by carbon monoxide, which binds to the reduced heme *a*_3_. The redox state of the enzyme as well as formation of the Cyt*c*O-CO complex were verified spectrophotometrically.

The Cyt*c*O-CO complex (with or without cyt. *c*) was mixed rapidly with an oxygen-saturated buffer (20 mM HEPES (pH 7.5), 0.05% DDM, 0.1 mM EDTA) at a Cyt*c*O:O_2_-saturated solution ratio of 1:5) in a flow-flash apparatus (Applied Photophysics). The CO ligand was photo-dissociated from heme *a*_3_, about 200 ms after mixing, by means of a 10-ns laser flash (Quantel Brilliant B, Nd-YAG, 532 nm), enabling oxygen to bind to heme *a*_3_. The subsequent reaction of the reduced Cyt*c*O with oxygen was monitored as absorbance changes over time at different single wavelengths^45^.

## References

[1] Kaila VRI, Verkhovsky MI, Wikström M (2010). Proton-coupled electron transfer in cytochrome oxidase. Chem. Rev..

[2] Hosler JP, Ferguson-Miller S, Mills DA (2006). Energy transduction: Proton transfer through the respiratory complexes. Annual Review of Biochemistry.

[3] Yoshikawa S (2006). Proton pumping mechanism of bovine heart cytochrome c oxidase. Biochimica et Biophysica Acta - Bioenergetics.

[4] Namslauer A, Brzezinski P (2004). Structural elements involved in electron-coupled proton transfer in cytochrome c oxidase. FEBS Lett.

[5] Brzezinski P, Gennis RB (2008). Cytochrome c oxidase: exciting progress and remaining mysteries. J. Bioenerg. Biomembr..

[6] Brzezinski P, Ädelroth P (2006). Design principles of proton-pumping haem-copper oxidases. Curr Opin Struct Biol.

[7] Richter OMH, Ludwig B (2009). Electron transfer and energy transduction in the terminal part of the respiratory chain - Lessons from bacterial model systems. Biochimica et Biophysica Acta - Bioenergetics.

[8] Ferguson-Miller S, Hiser C, Liu J (2012). Gating and regulation of the cytochrome c oxidase proton pump. Biochimica et Biophysica Acta - Bioenergetics.

[9] Rich PR, Maréchal A (2013). Functions of the hydrophilic channels in protonmotive cytochrome c oxidase. Journal of the Royal Society Interface.

[10] Konstantinov AA (2012). Cytochrome c oxidase: Intermediates of the catalytic cycle and their energy-coupled interconversion. FEBS Lett..

[11] Blomberg MRA, Siegbahn PEM (2014). Proton pumping in Cytochrome c oxidase: Energetic requirements and the role of two proton channels. Biochimica et Biophysica Acta (BBA) - Bioenergetics.

[12] Popović DM, Leontyev IV, Beech DG, Stuchebrukhov AA (2010). Similarity of cytochrome c oxidases in different organisms. Proteins: Structure, Function and Bioinformatics.

[13] Von Ballmoos C, Ädelroth P, Gennis RB, Brzezinski P (2012). Proton transfer in *ba*_3_ cytochrome *c* oxidase from *Thermus thermophilus*. Biochim. Biophys. Acta.

[14] Wikström M, Sharma V, Kaila VRI, Hosler JP, Hummer G (2015). New perspectives on proton pumping in cellular respiration. Chem. Rev..

[15] Wikström M, Morgan JE (1992). The dioxygen cycle. Spectral, kinetic, and thermodynamic characteristics of ferryl and peroxy intermediates observed by reversal of the cytochrome oxidase reaction. J Biol Chem.

[16] Wikström M (1989). Identification of the electron transfers in cytochrome oxidase that are coupled to proton-pumping. Nature.

[17] Wikström M (1994). Mechanism of Proton Translocation by the Respiratory Oxidases - the Histidine Cycle. Biochim. Biophys. Acta-Bioenerg..

[18] Verkhovsky MI, Jasaitis A, Verkhovskaya ML, Morgan JE, Wikström M (1999). Proton translocation by cytochrome c oxidase. Nature.

[19] Bloch D (2004). The catalytic cycle of cytochrome c oxidase is not the sum of its two halves. Proc. Natl. Acad. Sci. USA.

[20] Wikström M, Verkhovsky MI (2006). Towards the mechanism of proton pumping by the haem-copper oxidases. Biochimica et Biophysica Acta - Bioenergetics.

[21] Belevich I, Bloch DA, Belevich N, Wikström M, Verkhovsky MI (2007). Exploring the proton pump mechanism of cytochrome c oxidase in real time. Proc. Natl. Acad. Sci. USA.

[22] Sharma V, Karlin KD, Wikström M (2013). Computational study of the activated OH state in the catalytic mechanism of cytochrome c oxidase. Proc. Natl. Acad. Sci. USA.

[23] Blomberg MRA, Siegbahn PEM (2015). Protonation of the binuclear active site in cytochrome c oxidase decreases the reduction potential of Cu B. Biochimica et Biophysica Acta - Bioenergetics.

[24] Jancura D (2006). Spectral and kinetic equivalence of oxidized cytochrome c oxidase as isolated and “activated” by reoxidation. J. Biol. Chem..

[25] Brand SE (2007). A new ruthenium complex to study single-electron reduction of the pulsed OH state of detergent-solubilized cytochrome oxidase. Biochemistry.

[26] Hill BC (1994). Modeling the sequence of electron transfer reactions in the single turnover of reduced, mammalian cytochrome c oxidase with oxygen. J Biol Chem.

[27] Hill BC (1991). The reaction of the electrostatic cytochrome c-cytochrome oxidase complex with oxygen. J. Biol. Chem..

[28] Hirota S (1996). A flash-photolysis study of the reactions of a caa3-type cytochrome oxidase with dioxygen and carbon monoxide. J Bioenerg Biomembr.

[29] Brzezinski P, Larsson G (2003). Redox-driven proton pumping by heme-copper oxidases. Biochim. Biophys. Acta.

[30] Einarsdóttir Ó (1995). Fast Reactions of Cytochrome-Oxidase. Biochim. Biophys. Acta.

[31] Karpefors M, Ädelroth P, Zhen Y, Ferguson-Miller S, Brzezinski P (1998). Proton uptake controls electron transfer in cytochrome c oxidase. Proc. Natl. Acad. Sci. USA.

[32] Liao GL, Palmer G (1996). The reduced minus oxidized difference spectra of cytochromes a and a3. Biochim. Biophys. Acta.

[33] Vanneste WH (1966). The stoichiometry and absorption spectra of components a and a-3 in cytochrome c oxidase. Biochemistry.

[34] Szundi I, Liao GL, Einarsdóttir O (2001). Near-infrared time-resolved optical absorption studies of the reaction of fully reduced cytochrome c oxidase with dioxygen. Biochemistry.

[35] Gorbikova EA, Vuorilehto K, Wikström M, Verkhovsky MI (2006). Redox titration of all electron carriers of cytochrome c oxidase by Fourier transform infrared spectroscopy. Biochemistry.

[36] Sousa FL (2008). Redox properties of thermus thermophilusba3: Different electron-proton coupling in oxygen reductases?. Biophys. J..

[37] Melin F (2016). The unusual redox properties of C-type oxidases. Biochimica et Biophysica Acta - Bioenergetics.

[38] Alric J (2004). Electrostatic interaction between redox cofactors in photosynthetic reaction centers. J. Biol. Chem..

[39] Öjemyr LN, Von Ballmoos C, Faxén K, Svahn E, Brzezinski P (2012). The membrane modulates internal proton transfer in cytochrome c oxidase. Biochemistry.

[40] Näsvik Öjemyr L, Lee HJ, Gennis RB, Brzezinski P (2010). Functional interactions between membrane-bound transporters and membranes. Proc Natl Acad Sci USA.

[41] Wittenberg BA, Wittenberg JB (1989). Transport of oxygen in muscle. Annual Review of Physiology.

[42] Wikström M (2012). Active site intermediates in the reduction of O 2 by cytochrome oxidase, and their derivatives. Biochimica et Biophysica Acta - Bioenergetics.

[43] Mitchell DM, Gennis RB (1995). Rapid purification of wildtype and mutant cytochrome c oxidase from Rhodobacter sphaeroides by Ni(2+)-NTA affinity chromatography. FEBS Lett..

[44] Zhen Y (1998). Overexpression and purification of cytochrome coxidase from Rhodobacter sphaeroides. Protein Expression and Purification. Protein Expr. Purif..

[45] Brändén M (2001). On the role of the K-proton transfer pathway in cytochrome c oxidase. Proc. Natl. Acad. Sci. USA.

[46] Van Gelder BF, Slater EC (1962). The extinction coefficient of cytochrome c. BBA - Biochimica et Biophysica Acta.

[47] Boelens R, Wever R (1980). Redox reactions in mixed-valence cytochrome c oxidase. FEBS Lett..

